# Relative Fat Mass Associated with Hyperuricemia in Adults: A Cross-Sectional Study

**DOI:** 10.2174/0118715303344427241218114648

**Published:** 2025-02-06

**Authors:** Tian Gu, Zhaoxiang Wang, Qichao Yang, Mengjiao Xu, Xuejing Shao, Bingshuang Xue

**Affiliations:** 1 Department of Endocrinology, Affiliated Wujin Hospital of Jiangsu University, Wujin Clinical College of Xuzhou Medical University, Changzhou, Jiangsu, 213017, China;; 2 Department of Endocrinology, Affiliated Kunshan Hospital of Jiangsu University, Kunshan, Jiangsu, China

**Keywords:** Obesity, hyperuricemia, relative fat mass, NHANES, cross-sectional study, nonlinear relationship

## Abstract

**Aims:**

There is a close relationship between obesity and hyperuricemia. Relative Fat Mass (RFM) is considered a new indicator for evaluating obesity. We aim to explore the relationship between RFM and the risk of hyperuricemia in adults.

**Methods:**

This cross-sectional study included adult participants from the 2007-2018 National Health and Nutrition Examination Survey (NHANES). The RFM was calculated as: RFM =64 - (20 × height/waist circumference) + (12 × sex), where sex is defined as 0 for men and 1 for women. Hyperuricemia was confirmed by using serum uric acid (SUA) levels ≥ 7 mg/dL in men and ≥ 6 mg/dL in women. The relationship between RFM and the risk of hyperuricemia was thoroughly investigated.

**Results:**

A total of 29369 participants were enrolled in this study. The RFM levels in the hyperuricemia group were higher than those in the non-hyperuricemia group (*P* < 0.01). Logistic and linear regression indicated that RFM levels were positively associated with the risk of hyperuricemia (OR=1.08, 95% CI: 1.05-1.11, *P* < 0.001) and SUA levels (β=0.04, 95% CI: 0.03-0.05, *P* < 0.001). The relationship remained consistent across subgroups. Smooth curve fitting showed a nonlinear relationship, with an inflection point at 34.22. Above this threshold, the link between RFM levels and hyperuricemia was found to be more remarkable.

**Conclusion:**

Higher RFM is associated with an increased risk of hyperuricemia. RFM could act as a cost-efficient and straightforward measure for hyperuricemia risk assessment.

## 1 INTRODUCTION

Uric acid is the final product of purine analogue metabolism [1]. Hyperuricemia is the condition of having serum uric acid (SUA) levels above the normal range. Modern evidence-based research confirms that hyperuricemia is an early indicator and the primary cause of gout, and it has strong associations with components of metabolic syndrome [2, 3]. It is considered a significant risk factor for hypertension, diabetes, cardiovascular diseases, chronic kidney disease, and increased mortality [4-7]. The global incidence of hyperuricemia is growing, posing a significant burden worldwide.

Obesity is a major global health issue affecting billions worldwide [8]. The excessive accumulation of adipose tissue is closely associated with various health problems, including cardiovascular diseases, metabolic disorders, and cancers [9]. Although the Body Mass Index (BMI) is widely used, it has limitations in accurately assessing obesity, as it fails to reflect the proportions of bone, muscle, and fat mass, as well as gender differences [10]. While advanced assessment methods like computed tomography (CT) and magnetic resonance imaging (MRI) exist, their high cost, limited accessibility, time consumption, and radiation issues constrain their clinical application [11]. Thus, researchers have developed Relative Fat Mass (RFM), a new obesity indicator that uses Waist Circumference (WC) and height to better assess fat mass [12, 13]. Compared to BMI, when the Dual-energy X-ray Absorptiometry (DXA) method is used as the reference standard for diagnosing adult obesity, RFM demonstrates a lower rate of obesity misclassification [13]. Numerous studies have indicated that RFM is more closely associated with the risk of various diseases, such as hypertension, cardiovascular diseases, non-alcoholic fatty liver disease (NAFLD), type 2 diabetes, and depression, compared to BMI and WC [14-18].

It is well known that obesity is closely linked to hyperuricemia [19, 20]. However, the relationship between RFM and the risk of hyperuricemia remains unclear. Therefore, this study aims to explore the association between RFM and hyperuricemia using data from the National Health and Nutrition Examination Survey (NHANES) [21].

## 2 MATERIALS AND METHODS

### 2.1 Study Population

The dataset was derived from six consecutive NHANES cycles during 2007–2018. NHANES is a nationally representative survey assessing the health and nutrition of the US population (https://www.cdc.gov/nchs/nhanes/) [22]. The study was approved by the Research Ethics Review Board of the National Centre for Health Statistics (https://www.cdc.gov/nchs/nhanes/irba98.htm), and all participants provided written consent. This study comprised 29369 eligible participants, all of whom were 20 years or older, not pregnant, and had complete RFM and SUA data.

### 2.2 Exposure and Outcome Definitions

The RFM was utilized as the exposure variable, calculated using the formula proposed by Orison O. Woolcott and colleagues [12]. For males, it is expressed as RFM_male_ = 64 - [20 x 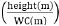], whereas for females, it is RFM_female_ = 76 - [20 x 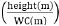]. On the other hand, hyperuricemia is identified by SUA levels reaching or exceeding 7 mg/dL in males and 6 mg/dL in females [23].

### 2.3 Covariate Definitions

This study accounted for confounding variables, such as demographics (age, gender, race), marital status, annual household income, education, smoking status, diabetes, hypertension, cardiovascular disease, and measurements of BMI, height, WC, alanine aminotransferase (ALT), aspartate aminotransferase (AST), triglyceride (TG), total cholesterol (TC), high-density lipoprotein cholesterol (HDL-c), low-density lipoprotein cholesterol (LDL-c), serum creatinine (SCr), and estimated glomerular filtration rate (eGFR). eGFR was calculated using the Chronic Kidney Disease Epidemiology Collaboration (CKD-EPI) formula, incorporating age, gender, race, and SCr levels [24]. Smoking status included both former and current smokers. Diabetes and hypertension were defined by self-reported history. Cardiovascular disease was identified by self-reported heart attack, stroke, heart failure, coronary artery disease, or angina.

### 2.4 Statistical Analysis

The statistical analyses followed the Centers for Disease Control and Prevention guidelines, using a complex multistage cluster survey design and weights from six cycles. Continuous variables were presented as means with standard errors (SE) and categorical variables as percentages. The weighted Student’s t-test and chi-squared test were used to compare continuous and categorical variables across groups. Weighted logistic and linear regression models were employed to investigate the associations between RFM (both as a continuous variable and by quartiles) and hyperuricemia, as well as SUA levels. Subgroup analysis was conducted based on age, gender, BMI, diabetes, hypertension, cardiovascular disease, and eGFR. Weighted smooth curve fitting analysis further investigated the relationship between RFM and hyperuricemia risk. For observed non-linear correlations, a two-piecewise linear regression model was used to identify the threshold point. All statistical analyses were performed using Empower software (http://www.empowerstats .com) and R software (http://www.R-project.org) [21]. A *P*-value < 0.05 was considered statistically significant.

## 3 RESULTS

### 3.1 Baseline Characteristics

This study comprised 29369 participants with an average age of 47.47 years (Table **1**). Compared to the non-hyperuricemia group, the hyperuricemia group was characterized by older age, a higher proportion of males (68.71% *vs.* 45.33%), a greater number of individuals with annual incomes below $20,000 (14.85% *vs.* 13.62%), more smokers (48.90% *vs.* 43.62%), and higher incidence of diabetes (14.55% *vs.* 8.63%), hypertension (50.00% *vs.* 28.23%), and cardiovascular diseases (13.36% *vs.* 7.27%) (*P* < 0.05). The hyperuricemia group also demonstrated elevated BMI, height, WC, ALT, AST, TG, TC, LDL-c, SCr, and SUA levels (*P* < 0.001). Conversely, a lower percentage of individuals in the hyperuricemia group had education above high school, with lower eGFR and HDL-c levels (*P* < 0.001). Significant differences in racial distribution were observed (*P* < 0.001). The RFM levels were notably lower in the hyperuricemia group than in the non-hyperuricemia group (*P* = 0.005).

### 3.2 Association Between the RFM and the Risk of Hyperuricemia

**Fig. (1):**
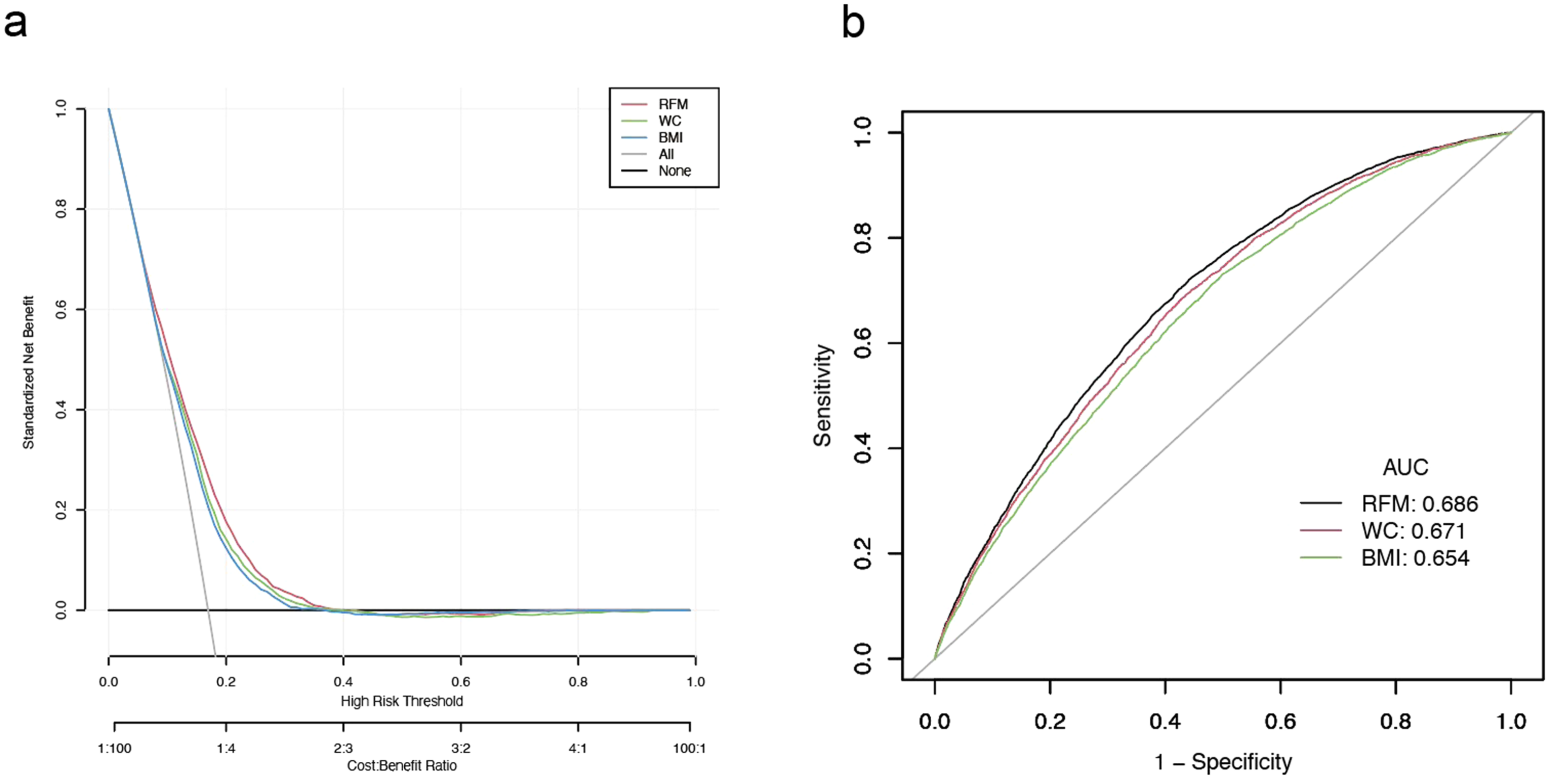
The results of DCA and ROC analyses (**a**, the results of DCA analysis; **b**, the results of ROC analysis).

The relationship between RFM and hyperuricemia prevalence remains statistically significant across adjusted Model 1 (OR=1.14, 95% CI: 1.13-1.15, *P* < 0.001), Model 2 (OR=1.13, 95% CI: 1.12-1.13, *P* < 0.001), and Model 3 (OR=1.08, 95% CI: 1.05-1.11, *P* < 0.001) (Table **2**). Based on RFM quantiles, participants were divided into four groups. Participants in the highest RFM quartile faced a 1.87-fold higher risk compared to those in the lowest quartile (OR=2.87, 95% CI: 1.96-4.19, *P* < 0.001). Additionally, linear regression analysis also indicated a significant relationship between RFM and SUA levels in the fully adjusted model (β=0.04, 95% CI: 0.03-0.05, *P* < 0.001) (Table **3**). Based on the results of DCA and ROC analysis, RFM demonstrated superior performance compared to traditional obesity indices (Figs. **1a** and **b**). The corresponding area under the curve (AUC) is as follows: RFM (68.6%), WC (67.1%), and BMI (65.4%) (Fig. **1b**).

### 3.3 Smooth Curve Fitting and Threshold Effect

**Fig. (2):**
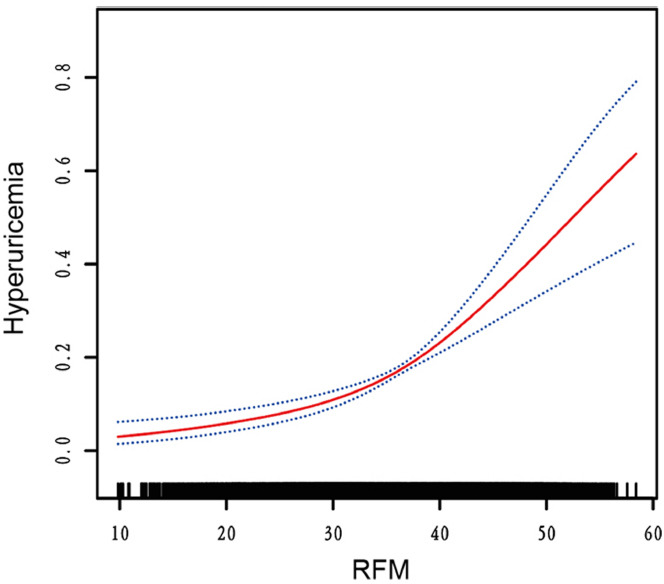
The results of smooth curve fitting analysis.

We conducted a smooth curve fitting analysis on the relationship between RFM and hyperuricemia and observed a distinct inflection point between the two (*P* for nonlinearity<0.001). The two-piecewise linear regression model further identified a breakpoint for the overall population's RFM level at 34.22 (Fig. **2** and Table **4**). An increase in RFM levels correlates with a rise in hyperuricemia risk, characterized by an OR of 1.07 and a 95% CI ranging from 1.05 to 1.10. Beyond this threshold, as RFM levels exceed 34.22, the association between RFM levels and hyperuricemia risk becomes more remarkable (OR=1.11, 95% CI: 1.07-1.15, *P* < 0.001).

### 3.4 Subgroup Analysis

**Fig. (3):**
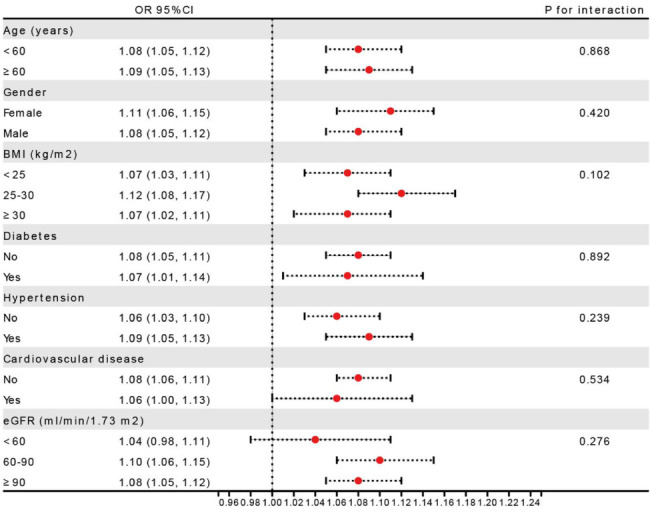
The results of the subgroup analysis.

We performed subgroup analyses stratified by factors, such as age, gender, BMI, diabetes, hypertension, cardiovascular disease, and eGFR (Fig. **3**). The results showed no significant variation in the RFM-hyperuricemia association among these groups (*P* for interaction > 0.05).

## 4 DISCUSSION

This novel research focused on the relationship between RFM and hyperuricemia in a large population sample. Both logistic and linear models demonstrated a strong association between RFM and hyperuricemia, as well as SUA levels. Smooth curve fitting revealed a nonlinear relationship, identifying a threshold point at 34.22. Above this threshold, this relationship was found to be more remarkable. Additionally, RFM outperformed traditional obesity markers in its specificity toward hyperuricemia risk.

As previously stated, the “obesity paradox” continues to pose challenges in epidemiological studies due to the intricate relationships between various body measurement indices, complicating the precise identification of biologically driven disease risks [25, 26]. Individuals with identical BMI can exhibit substantial differences in body shape and fat distribution, necessitating a clear distinction between lean mass and fat mass [27]. The RFM, derived through a linear equation involving height and WC, has been extensively validated across diverse populations [12, 28]. Earlier research has emphasized the efficacy of RFM in accurately evaluating body composition and forecasting disease risk [29, 30]. In contrast to more intricate emerging obesity indices, RFM is straightforward to calculate, making regular screening practical for the general populace. Studies in epidemiology demonstrate that obesity is a major risk factor for hyperuricemia, as its prevalence is higher in obese populations [31, 32]. Likewise, individuals with hyperuricemia are at a greater risk of obesity than those with normal uric acid levels. This suggests a close interrelationship between obesity and hyperuricemia [33, 34]. Previous studies have also identified a connection between RFM and uric acid levels in children suffering from chronic kidney disease [35]. Given the significant differences in fat distribution between children and adults, it is essential to assess the relevance of RFM as an obesity indicator across various groups. This research distinctively examined this correlation among the general adult population in the U.S. As previously emphasized in research on RFM, it was found that RFM demonstrates superior performance in assessing the risk of hyperuricemia compared to BMI and WC. Some potential biological mechanisms help explain the association between RFM and hyperuricemia. The interplay between lipid metabolism and uric acid metabolism is evident [36]. Higher uric acid levels decrease lipoprotein lipase activity, impacting lipid processing and altering adipokine levels, resulting in fat redistribution and potential obesity [2, 37]. Additionally, uric acid also provokes inflammatory and oxidative responses in adipocytes [38]. It activates the nuclear factor kappa B (NF-κB) signalling pathway, leading to the release of pro-inflammatory cytokines, such as tumour necrosis factor-alpha (TNF-α) and interleukin-6 (IL-6) [39-41]. These inflammatory responses can exacerbate insulin resistance and contribute to metabolic disorders. Additionally, uric acid increases the production of reactive oxygen species (ROS), resulting in oxidative stress that impairs adipocyte function and further disrupts metabolic homeostasis [42, 43]. On the other hand, adipocytes function as key endocrine organs, releasing hormones like visfatin, leptin, and adiponectin [44, 45]. Visfatin and leptin are linked to insulin resistance and inflammation, potentially increasing uric acid levels [46, 47]. Adiponectin, with its anti-inflammatory and insulin-sensitizing effects, is usually associated with reduced uric acid levels [48]. These hormones influence uric acid metabolism by affecting insulin sensitivity, inflammation, and kidney function, either directly or indirectly [49-51].

There are also some limitations to this study. First, due to its cross-sectional design, a future prospective study with a larger sample size is required to better understand causal relationships. Second, although some potential covariates were adjusted, the possibility of other confounding factors, such as metabolic syndrome, NAFLD, and medication history, influencing the results cannot be entirely excluded. Lastly, this study focused on adults in the United States; the correlation between RFM and hyperuricemia risk in other populations needs further investigation.

## CONCLUSION

This representative national research presents initial evidence of a positive correlation between RFM and hyperuricemia. RFM could be utilized as a simple and affordable biomarker for determining the risk of hyperuricemia.

## Figures and Tables

**Table 1:** Baseline characteristics of the study population.

-	Overall (N = 29369)	Non- hyperuricemia (N = 24396)	Hyperuricemia (N = 4973)	*P* value
Age (years)	47.47Añ0.23	46.92Añ0.24	50.34Añ0.32	<0.001
Gender, %	-	-	-	<0.001
Male	49.06	45.33	68.71	
Female	50.94	54.67	31.29	
Married, %	55.59	55.66	55.19	0.553
Race, %	-	-	-	<0.001
Mexican American	8.64	9.00	6.75	-
Non-Hispanic Black	10.55	10.22	12.27	-
Non-Hispanic White	66.90	66.60	68.45	-
Other Hispanic	5.93	6.21	4.49	-
Other Races	7.98	7.97	8.04	-
Annual household income (below $20,000), %	13.82	13.62	14.85	0.028
Education level (above high school), %	61.46	61.95	58.91	<0.001
Smokers, %	44.47	43.62	48.90	<0.001
Diabetes, %	9.58	8.63	14.55	<0.001
Hypertension, %	31.71	28.23	50.00	<0.001
Cardiovascular disease, %	8.25	7.27	13.36	<0.001
BMI (kg/m^2^)	29.00Añ0.09	28.38Añ0.09	32.31Añ0.16	<0.001
Height (cm)	168.65Añ0.11	168.14Añ0.11	171.29Añ0.22	<0.001
WC (cm)	99.33Añ0.22	97.52Añ0.23	108.85Añ0.39	<0.001
ALT (U/L)	25.27Añ0.15	24.07Añ0.13	31.57Añ0.51	<0.001
AST (U/L)	25.22Añ0.12	24.54Añ0.12	28.80Añ0.33	<0.001
TG (mmol/L)	1.40Añ0.02	1.32Añ0.02	1.78Añ0.04	<0.001
TC (mmol/L)	4.99Añ0.01	4.97Añ0.01	5.11Añ0.02	<0.001
HDL-c (mmol/L)	1.38Añ0.01	1.41Añ0.01	1.24Añ0.01	<0.001
LDL-c (mmol/L)	2.94Añ0.01	2.93Añ0.01	3.02Añ0.03	<0.001
Scr (I1/4mol/L)	78.02Añ0.24	75.39Añ0.24	91.79Añ0.55	<0.001
eGFR (ml/min/1.73 m^2^)	94.77Añ0.31	96.70Añ0.32	84.62Añ0.47	<0.001
SUA (mg/dL)	5.43Añ0.01	5.00Añ0.01	7.66Añ0.02	<0.001
RFM	35.30Añ0.09	35.24Añ0.10	35.62Añ0.17	0.005

**Abbreviations:** BMI, body mass index; WC, waist circumference; ALT, alanine transaminase; AST, aspartate transaminase; TG, triglyceride; TC, total cholesterol; HDL-c, high-density lipoprotein cholesterol; LDL-c, low-density lipoprotein cholesterol; SUA, serum uric acid; Scr, serum creatinine; eGFR, estimated glomerular filtration rate; RFM, relative fat mass.

**Table 2:** Logistic regression analysis results of RFM and hyperuricemia.

Hyperuricemia	OR (95%CI) *P* Value		
-	**Model 1**	**Model 2**	**Model 3**
**Continuous**			
RFM	1.14 (1.13, 1.15) <0.001	1.13 (1.12, 1.13) <0.001	1.08 (1.05, 1.11) <0.001
**Categories**			
Quantile 1	Reference	Reference	Reference
Quantile 2	2.11 (1.93, 2.31) <0.001	1.97 (1.80, 2.17) <0.001	1.29 (1.10, 1.52) 0.002
Quantile 3	3.94 (3.50, 4.43) <0.001	3.40 (3.01, 3.85) <0.001	1.75 (1.36, 2.25) <0.001
Quantile 4	14.88 (12.62, 17.54) <0.001	11.50 (9.67, 13.69) <0.001	2.87 (1.96, 4.19) <0.001
*P* for trend	<0.001	<0.001	<0.001

**Note: ** 95% CI: 95% confidence interval.

Model 2: adjusted for age, gender, and race.

Model 3: adjusted for age, gender, race, marital status, annual household income, education level, smoking history, diabetes, hypertension, cardiovascular disease, ALT, AST, TG, HDL-c, LDL-c, Scr, and eGFR.

**Abbreviation:** OR: Odds Ratio.

**Table 3:** Linear regression analysis results of RFM and SUA levels.

SUA	β (95%CI) *P*-Value			
-	Model 1	Model 2	Model 3	
**Continuous**				
RFM	0.08 (0.08, 0.08) <0.001	0.07 (0.07, 0.08) <0.001	0.04 (0.03, 0.05) <0.001	
**Categories**				
Quantile 1	Reference	Reference	Reference	
Quantile 2	0.46 (0.41, 0.50) <0.001	0.41 (0.37, 0.45) <0.001	0.10 (0.04, 0.17) 0.002	
Quantile 3	0.86 (0.80, 0.91) <0.001	0.79 (0.73, 0.85) <0.001	0.21 (0.11, 0.31) <0.001	
Quantile 4	1.60 (1.53, 1.66) <0.001	1.47 (1.41, 1.54) <0.001	0.46 (0.32, 0.60) <0.001	
*P* for trend	<0.001	<0.001	<0.001	

**Note: ** 95% CI: 95% confidence interval.

Model 2: adjusted for age, gender, and race.

Model 3: adjusted for age, gender, race, marital status, annual household income, education level, smoking history, diabetes, hypertension, cardiovascular disease, ALT, AST, TG, HDL-c, LDL-c, Scr, and eGFR.

**Table 4:** Threshold effect analysis of RFM on hyperuricemia risk.

Model	OR (95% CI) *P* value
**Total**	-
Breakpoint (K)	34.22
OR1 (<34.22)	1.07 (1.05, 1.10) <0.001
OR2 (>34.22)	1.11 (1.07, 1.15) <0.001
OR2/OR1	1.04 (1.01, 1.06) 0.011
*P* for logarithmic likelihood ratio	0.011

## Data Availability

The data are sourced from the NHANES database, which is a publicly accessible and free resource (https://www.cdc.gov/nchs/nhanes). The summary data supporting the findings of this study are available upon request from the corresponding author.

## LIST OF ABBREVIATIONS

NHANES: National Health and Nutrition Examination Survey
SUA: Serum Uric Acid
BMI: Body Mass Index
CT: Computed Tomography
MRI: Resonance Imaging
RFM: Relative Fat Mass
WC: Waist Circumference
DXA: Dual-energy X-ray Absorptiometry

## STANDARDS OF REPORTING

STROBE guidelines were followed.
